# Identifying risk factors for healthcare-associated infections from electronic medical record home address data

**DOI:** 10.1186/1476-072X-9-47

**Published:** 2010-09-17

**Authors:** Jeffrey S Wilson, David C Shepherd, Marc B Rosenman, Abel N Kho

**Affiliations:** 1 Department of Geography, Indiana University - Purdue University Indianapolis, Indianapolis, Indiana, USA; 2 School of Medicine, Indiana University, Indianapolis, Indiana, USA; 3 The Regenstrief Institute, Inc., Indianapolis, Indiana, USA; 4 Feinberg School of Medicine, Northwestern University, Chicago, Illinois, USA

## Abstract

**Background:**

Residential address is a common element in patient electronic medical records. Guidelines from the U.S. Centers for Disease Control and Prevention specify that residence in a nursing home, skilled nursing facility, or hospice within a year prior to a positive culture date is among the criteria for differentiating healthcare-acquired from community-acquired methicillin-resistant *Staphylococcus aureus *(MRSA) infections. Residential addresses may be useful for identifying patients residing in healthcare-associated settings, but methods for categorizing residence type based on electronic medical records have not been widely documented. The aim of this study was to develop a process to assist in differentiating healthcare-associated from community-associated MRSA infections by analyzing patient addresses to determine if residence reported at the time of positive culture was associated with a healthcare facility or other institutional location.

**Results:**

We identified 1,232 of the patients (8.24% of the sample) with positive cultures as probable cases of healthcare-associated MRSA based on residential addresses contained in electronic medical records. Combining manual review with linking to institutional address databases improved geocoding rates from 11,870 records (79.37%) to 12,549 records (83.91%). Standardization of patient home address through geocoding increased the number of matches to institutional facilities from 545 (3.64%) to 1,379 (9.22%).

**Conclusions:**

Linking patient home address data from electronic medical records to institutional residential databases provides useful information for epidemiologic researchers, infection control practitioners, and clinicians. This information, coupled with other clinical and laboratory data, can be used to inform differentiation of healthcare-acquired from community-acquired infections. The process presented should be extensible with little or no added data costs.

## Background

Information on patient residential location can inform epidemiologic research and clinical decisions for some types of infections. For example, residence in long-term care facilities, including nursing homes and rehabilitation hospitals, is a risk factor for healthcare-associated methicillin-resistant *Staphylococcus aureus *(HA-MRSA) infection [1-3]. Risk factors for community-associated methicillin-resistant *Staphylococcus aureus *(CA-MRSA) infection include residence in a shelter, military barracks, or correctional facility [4-9]. Differentiating between these two types of multi-drug resistant infections can guide healthcare decisions and inform epidemiologic and public health analyses. Patient home address data in electronic medical record systems (EMRs) may be useful for classifying residence type, but methods of residential categorization based on EMR addresses have not been widely documented.

Our objective was to develop a baseline process to assist in differentiating HA-MRSA from CA-MRSA infections using patient home address data from an EMR. This work was completed as part of a larger study aimed at predicting hospitalization resulting from CA-MRSA. One of the initial steps in the larger study was to analyze a retrospective cohort of patients infected or colonized with MRSA to distinguish cases of HA-MRSA from CA-MRSA. Current guidelines from the U.S. Centers for Disease Control and Prevention (CDC) specify that residence in a nursing home, skilled nursing facility, or hospice within the previous year is among the criteria for differentiating HA-MRSA from CA-MRSA [10]. Thus, to meet the CDC case definition for CA-MRSA, we needed to exclude patients residing in healthcare facilities.

We also sought to identify cases of MRSA associated with non-healthcare institutional addresses, including shelters and correctional facilities, to detect potential clusters of infection at these locations. In addition to identifying residence type, we developed a spatial database of patient residential locations to facilitate future analysis of spatiotemporal infection clustering. The processes we used were based on geocoding (matching textual address information to spatial locations) using geographic information system (GIS) software.

## Methods

### Patient Data

Patient records indicating positive MRSA infection or colonization were extracted from the Indiana Network for Patient Care (INPC). The INPC is a regional health information organization that collects medical data from five major hospital systems, including fifteen hospital facilities and multiple clinics in metropolitan Indianapolis [11]. Since 2006, infection control providers have entered information on MRSA cases into the INPC [12]. We queried the INPC databases for patients with infection control entries indicating MRSA infection or colonization from January 1, 2006 to March 10, 2009. A total of 14,956 individual patients were identified. We extracted the street name and number, city, state, and ZIP code stored in the EMR as of the date when the infection control providers entered the first positive MRSA culture for these patients.

While additional patient-specific information is available through the EMR, we only extracted addresses and culture dates for this study to lessen the potential for compromising patient confidentiality. All data processing was conducted on secure servers and individual patients were assigned a unique random identification number. Approval for this study was granted by the Indiana University Institutional Review Board.

### Institutional Residential Facilities

We obtained two publicly-available databases of nursing homes in the state of Indiana. The Nursing Home Compare database, available from the U.S. Department of Health and Human Services (HHS), contains information for facilities that provide skilled nursing or rehabilitation services and are certified to participate in Medicare/Medicaid [13]. The HHS database contained names and addresses for 511 nursing homes in the state. State health departments can license additional nursing homes that are not listed in the HHS database. The Indiana State Department of Health's (ISDH) Comprehensive Care Facility Licensing and Certification Program database contained addresses for 611 facilities in Indiana [14]; 145 of these facilities were not included in the HHS database. In total, facility names and addresses for 656 unique nursing homes were identified by combining the state and federal databases.

Freely-accessible state and federal databases were also used to obtain names and addresses for hospitals in Indiana. The HHS Hospital Compare database [15] listed 115 facilities in the state, but was limited to facilities that report quality information to HHS. The ISDH Hospital Licensing and Certification Program databases contained names and addresses for 165 hospitals, including all of those listed in the HHS database [16]. Hospitals unique to the ISDH database were classified as long-term, psychiatric, or rehabilitation facilities. All the hospitals listed in the HHS Hospital Compare Database for Indiana were classified as acute care or critical access facilities.

Addresses for 20 adult and 7 juvenile correctional facilities operated by the Indiana Department of Corrections were collected from that organization's website [17]. We obtained information for federal correctional facilities from the U.S. Department of Justice, but we later learned that none of the patient addresses matched to these locations. We were unable to find comprehensive, publicly-available databases for correctional facilities at the local (county and city) level. However, using local government websites, we obtained addresses and names for jails in the nine-county Indianapolis metropolitan area. A database listing 151 shelters and transitional housing facilities in the state was obtained from the Indiana Housing and Community Development Authority [18].

### Geocoding

Automated street address matching routines in a commercial GIS software package (ArcGIS 9.3.1, ESRI, Redlands, CA) were used to process patient home addresses in the current study. The basic address matching process in ArcGIS begins with parsing addresses into individual components including street name, street prefix and suffix (e.g., West or Ave.), building number, city, state, and ZIP code. Prefixes and suffixes are standardized and the addresses are then compared to the reference data (ESRI Street Map 2005 data were used in the current study). A geocoding score is assigned to each potential match and the candidate with the highest geocoding score is then used to assign a point location for each record using linear interpolation along a street segment. We used the default parameter settings for minimum match scores and spelling sensitivity in the current study.

Geocoding was implemented in two sequential steps. In step 1, we geocoded the original patient addresses obtained from the EMR. Manual review of these results identified non-geocoded records that included a facility name in the address field but no street name or street number (e.g., ABC Nursing Home). Lack of street name and number in the address field prevented the geocoding algorithm from matching these records to a geographic location. However, these records potentially represented institutional residential locations and could be useful for differentiating probable cases of HA-MRSA from CA-MRSA.

In step 2, standardized patient addresses output from the first geocoding iteration were compared to nursing home, hospital, shelter, and correctional facility databases. The objective in this step was to identify patients residing in institutional facilities. We used both facility name and addresses in the institutional databases as potential items for matching with patient addresses in the EMR records. If a patient record matched to an institution based on a facility name, we repopulated the patient address fields with the street name and building number from the institutional database. For all patient records matching to an institution (based on either a facility name or address), we added new fields to the patient record to indicate the facility category (nursing home, hospital, shelter, or correctional facility). Additional manual review and editing of non-geocoded addresses was carried out by an analyst. This modified set of patient home addresses was put through a second geocoding iteration.

We compared the number of successfully geocoded records output from step 1 and step 2. The purpose of this comparison was to determine if improvement in geocoding rates result from linking patient records to institutional facility databases. We also compared the match rate between patient addresses and institutional databases using both the original address data from the EMR and standardized address data output from the first geocoding iteration. The purpose of this comparison was to determine if improvements in match rates between patient EMR records and institutional databases could be gained from the address standardization built into the geocoding process.

## Results

In the first geocoding iteration, 11,870 records (79.37%) were successfully geocoded using the original EMR address data. Manual review of non-geocoded records (*n *= 3,086) identified 266 patients (1.78%) with no address and 232 records (1.55%) with only a post office or rural route box number; these records were not amenable to automated geocoding with standard address matching methods. An additional 291 (1.95%) of the non-geocoded records included a facility name in the address field, indicating a potential institutional residential location. The remaining 2,297 (15.36%) records were not successfully geocoded in the first iteration because of data entry errors in the EMR address or errors/exclusions in the geographic reference layer used as a basis for geocoding (ESRI StreetMap 2007).

In the second geocoding iteration, after cleaning the patient address data by linking to institutional databases and manual editing, the number of successfully geocoded records increased to 12,549 (83.91%). We were able to geocode all 291 records that included a facility name. These records were unmatched in the first geocoding iteration because of the lack of street name and number. The reason these records were successfully geocoded in the second iteration was a result of adding street name and number obtained by linking these records to publicly-available institutional address data. An additional 388 records were successfully matched in the second iteration as a result of manual review and editing. Manual editing was implemented by reviewing each unmatched address and, where sufficient information was available, correcting mistakes or adding missing information. The most common problems noted for records that were corrected through manual review were misspellings of street names, missing or incorrect ZIP codes, and addresses associated with newer streets that were not included in the original reference layer. More current GIS street data provided by the City of Indianapolis provided information that enabled us to successfully geocode addresses in Marion County, IN where most of the patients resided.

**Figure 1:**
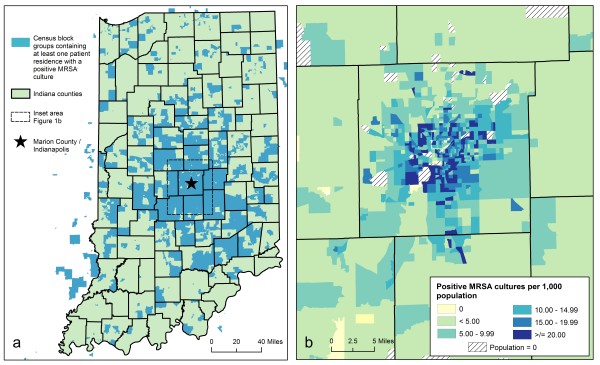
**Spatial patterns of patient residences with MRSA colonization or infection in the Indiana Network for Patient Care (INPC)**. Figure 1a depicts census block groups containing at least one patient residence with a positive MRSA culture. Figure 1b shows rates of positive MRSA cultures per 1,000 population by census block groups in Marion County/Indianapolis and surrounding areas.

Figure 1a shows census block groups in Indiana containing at least one geocoded residence associated with a MRSA infection or colonization identified in the INPC. As expected, cases were concentrated around central Indiana, proximal to INPC-affiliated hospital and clinic locations. Figure 1b depicts positive culture rates by census block group in Marion County (which shares a common boundary with the City of Indianapolis) and surrounding areas.

**Figure 2:**
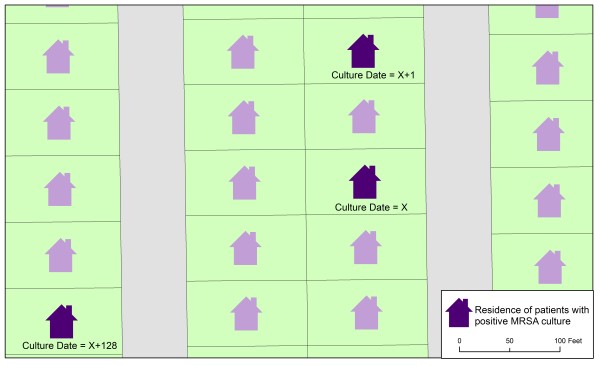
**Example of parcel-level geocoding of patients with positive MRSA cultures**. The figure shows a real example of geocoded MRSA cases, but the map orientation, parcel shapes, and dates have been generalized to protect confidentiality and lessen the potential for reverse geocoding.

Figure 2 illustrates the potential utility of examining geocoded patient addresses at a finer level of geographic detail. We are able to link patient home addresses to individual parcels within Indianapolis using GIS data from city planning offices. We observed multiple examples of MRSA-related patient visits that occurred within a short time span and were associated with residences in close geographic proximity. For example, two residences highlighted in the center of Figure 2 were associated with patients with positive culture dates occurring within one day of each other. These cases potentially represent an instance of MRSA transmission. Whether the patients had any interaction is unknown, but the example is illustrative of the potential for EMR-driven, GIS-based recognition of MRSA transmission patterns in the community.

Standardization of EMR patient addresses through the geocoding process increased the number of records that matched to institutional residential locations. When comparing the original patient addresses from the EMR to institutional databases, 545 records (3.64%) were successfully matched. Repeating the matching procedure using standardized addresses output from the geocoding procedure increased the number of institutional matches to 1,379 (9.22%).

Table 1 summarizes the final results. Matches to healthcare-associated facilities (nursing homes and hospitals) totalled 1,232 (8.24%). Nursing homes accounted for the largest proportion of patients associated with healthcare facilities (*n *= 1,193, 7.98%). A majority of these nursing homes were included in the HHS Nursing Home Compare database; only 73 records were associated with a nursing home unique to state health department databases. We found 39 (0.26%) patient records matching to a hospital and nearly half of these were associated with long-term, psychiatric or rehabilitation hospitals unique to state health department databases. A relatively small number of records matched to correctional institutions (*n *= 106, 0.71%) or shelters (*n *= 41, 0.27%).

**Table 1:** Patients with positive MRSA cultures matching to institutional address databases

Residential Category	# of Patients	% of Total Records
**Nursing Home**	**1,193**	**7.98%**
*HHS Database*	*1,120*	*7.49%*
*ISDH Database*	*73*	*0.49%*

**Hospital**	**39**	**0.26%**
*HHS Database*	*22*	*0.15%*
*ISDH Database*	*17*	*0.11%*

**Subtotal Healthcare-Associated**	**1,232**	**8.24%**

**Other Institution**	**147**	**0.98%**
*Shelter*	*41*	*0.27%*
*Correctional Facility*	*106*	*0.71%*

**Overall Total Institutional**	**1,379**	**9.22%**

## Discussion

Patient addresses in EMRs can be linked to institutional address data in standard database programs using SQL queries (e.g., ABC = ABC), but there are at least two advantages to using a geocoding approach and GIS software. First, geographic coordinates are returned for addresses that are successfully geocoded. This information can be useful for visual or analytic interpretation of spatiotemporal patterns of infection. Previous studies have illustrated the use of geocoded data to identify clusters of CA-MRSA in Chicago, IL, [19] Springfield, MA, [20] and West Midlands, UK [21]. Second, geocoding incorporates a standardization process that partitions address elements into different components (street name and number, street prefix and suffix, city, state, and ZIP code) and standardizes spelling and abbreviations. Even if the address does not geocode to a geographic location, the standardization process can facilitate improved record-to-record matching between EMRs and institutional address data. In our sample, address standardization more than doubled the number of patient addresses that we were able to associate with an institutional facility.

We used institutional name and address data that can be openly accessed via the Internet from federal, state, and local agencies. Therefore, the process we used in this study should be extensible with little or no added data costs. We found that the HHS Nursing Home Compare database accounted for the majority of patients associated with a nursing home facility, but a small percentage of nursing home matches were facilities unique to state health department databases. Conversely, while they were less frequent in number, about half of the patient records matching to a hospital were associated with facilities unique to state health department databases. This is understandable, given that the HHS Hospital Compare database focuses on acute care or critical access facilities, while state databases include long-term care hospitals.

Knowing that a patient resides, or has recently resided, in a healthcare-associated facility can affect healthcare decisions involving antibiotic therapy and infection control precautions. Given the increased incidence of multi-drug resistant organisms in such facilities, it would be advantageous to know a patient's residential location when initiating antibiotic therapy for a presumed infection. For instance, the rate of MRSA pneumonia is known to be higher in patients residing in long-term care facilities [22] as is the rate of *C. difficile *diarrhea [23]. Both pneumonia and diarrhea would be treated more aggressively knowing that a patient resides or has recently resided in a healthcare-associated facility. Likewise, until an infectious organism is identified, a physician may conservatively decide to implement infection control precautions.

Patient residence information may also be useful in biosurveillance to recognize outbreaks of infectious organisms in communities. Early detection of an evolving epidemic could provide an opportunity for intervention, mitigating the spread of infection. Further, such information could be used to more efficiently allocate educational resources to facilities and neighborhoods identified as "hot spots" for particular multi-drug resistant organisms.

The methods presented have several limitations. Some of the matches between patient records and healthcare facilities required manual editing due to variations in the way facility names were entered in the EMR. For example, companies may operate multiple nursing home facilities with similar names (e.g., "ABC Nursing Home South", "ABC Nursing Home North", etc.). If the EMR address contained only "ABC Nursing Home", matching to a specific facility required additional information, such as ZIP code. Misspellings of facility names presented similar problems that required manual editing. All of the automated matches we achieved were based on exact agreement between institutional and EMR patient databases. Future implementations of this method could investigate algorithms that permit some flexibility in matching requirements (e.g., fuzzy matching) but results should be reviewed to ensure that less than perfect matches do not produce false positives that can result from similarities in facility names. In addition, previous studies have documented geocoding biases that result in lower match rates in rural areas [24] and among disadvantaged and minority populations [25]. Prior to using geocoded data for analytical purposes, such as cluster analysis, these potential biases should be investigated. Despite these limitations, our process identified patient risk factors that were not readily apparent in existing EMR data and provided results that enabled us to more accurately identify a cohort of CA-MRSA patients.

Among the lessons learned from implementing this study is that there can be a substantial errors in EMR patient addresses that results at the point of collection (e.g., when a patient provides their home address at a physician's office or when the address is entered into the EMR). As EMRs are increasingly integrated with GIS, there is potential to improve the collection of address data at the point of capture. For example, just as a spell checking feature can highlight potential spelling errors in real time, technologies that integrate geographic information with patient data entry programs could highlight potential address errors while the data are being entered. Similarly, by cross checking patient address with geographic databases, potential errors can be highlighted after data entry that prompt requisitions for updated address data at follow-up contacts with the health care system.

## Conclusions

While residential location is only one piece of the puzzle, the methods presented contributed to more thorough differentiation of HA-MRSA from CA-MRSA cases based on CDC criteria. Recent federal legislation through the American Recovery and Reinvestment Act of 2009 promotes the extensive adoption of EMRs. The method we present in this paper provides a baseline methodology that can be applied to other EMRs in combination with freely-accessible databases of institutional residential facilities and geocoding tools that are available in most GIS software packages.

Enabling clinicians and public health practitioners to readily identify geographic risk factors may improve infection control practice and antibiotic choice. EMR address data collected as part of patient care can be used in novel ways to answer epidemiologic questions. Data on risk factors, such as prior healthcare exposure, may be time consuming and costly to obtain using manual chart review procedures. Finding ways to use the electronic records to obtain risk factor data could enhance the reuse of data and reduce the cost of retrospective epidemiologic studies.

## Competing interests

The authors declare that they have no competing interests.

## Authors' contributions

JSW conceived the general methodology, carried out the GIS analysis, and drafted the initial manuscript. DCS, ANK, and MBR contributed to conceptualizing the clinical and public health applications of the method, and provided background information on the Indiana Network for Patient Care. All authors contributed to writing and approved the final manuscript.
